# Mutation profiles of follicular thyroid tumors by targeted sequencing

**DOI:** 10.1186/s13000-019-0817-1

**Published:** 2019-05-10

**Authors:** Huanli Duan, Xiaoding Liu, Xinyu Ren, Hui Zhang, Huanwen Wu, Zhiyong Liang

**Affiliations:** Molecular Pathology Research Center, Department of Pathology, Peking Union Medical College Hospital, Chinese Academy of Medical Science, Peking Union Medical College, Beijing, 100730 China

**Keywords:** Follicular thyroid tumor, Targeted next generation sequencing, *TERT* promoter mutation

## Abstract

**Background:**

One of the major challenges remaining in the classification of thyroid tumor is the determination of whether a nodule is benign or malignant. We aimed to characterize the mutational profiles of follicular thyroid tumor and to identify markers with potential diagnostic and prognostic implications.

**Methods:**

Targeted sequencing with a panel of 18 thyroid cancer-related genes was performed on 48 tissue samples from follicular thyroid adenoma (FTA), 32 follicular tumors of uncertain malignant potential (FT-UMP), 17 well-differentiated tumors of uncertain malignant potential (WDT-UMP) and 53 samples from follicular thyroid carcinoma (FTC). The correlation of mutation profiles and clinicopathological features and prognosis were also analyzed.

**Results:**

We identified 95 nonsilent mutations spanning 14 genes. Specifically, *TERT* promoter *(TERTp)* mutations were exclusively detected in FTC. A total of 80% *EIF1AX* exon 2 mutations (4/5) and 75% *TSHR* mutations (3/4) occurred in FTA, whereas the rest of them occurred in FT-UMP. *KRAS* mutations and *TP53* mutations were only presented in borderline or malignant tumors. *H/N-RAS* mutations were detected in all four subtypes, but were most commonly found in WDT-UMP (*p* = 0.031). All *N-RAS* mutations were located at codon 61. *BRAF* V600E and *RET* fusion were absent in the entire cohort. In FTC cases, *EIF1AX* mutations were all located at intron 5/exon 6 and correlated with advanced disease (*p* = 0.032). Both *EIF1AX* and *TERTp* mutations predicted shorter disease-free survival (*p* = 0.007, *p* = 0.024, respectively). Further analysis revealed that *TERTp* mutations were correlated with shorter disease-free survival in patients with minimally invasive /encapsulated angioinvasive FTC (*p* = 0.017), but not in those with widely invasive FTC (*p* = 0.297).

**Conclusion:**

*TERTp*, *EIF1AX, TSHR, H/N/K-RAS* and *TP53* mutations may have diagnostic or prognostic potential in follicular thyroid tumors. *TERTp* mutations may predict a poor outcome in patients with minimally invasive/encapsulated angioinvasive FTC.

## Background

Thyroid cancer, the most common type of endocrine malignancy, predominantly arises from thyroid follicular cells, with approximately 95% being differentiated thyroid cancer. As one of the categories, follicular thyroid carcinoma (FTC) is a high-risk cancer with the likelihood of distant relapse [1].FTC is the malignant counterpart of follicular thyroid adenoma (FTA), the latter commonly found as benign neoplasm of the thyroid gland. Thyroid tumors with uncertain malignant potential (TT-UMP) are defined as the tumors presenting questionable capsular or vascular invasion and fail to meet the criteria for carcinoma, comprising follicular tumors with uncertain malignant nature (FT-UMP) and well-differentiated tumor of uncertain malignant potential (WDT-UMP) [2]. Currently, one of the major challenges remaining in the differentiation among FTC, TT-UMP and FTA is the interobserver variability in the histologic interpretation of capsular or vascular invasion, which largely depends on the liberty of pathologists, even in the surgically resected samples [3, 4].

*TERT* promoter (*TERTp*) mutations, firstly reported in both familial and sporadic melanomas, were regarded as important mechanisms contributing to increased telomerase activity in malignant cells [5]. Recent studies showed that in FTC, the frequency of *TERTp* mutations ranges from 14 to 36% [6–12]. Numerous studies have shown that *TERTp* mutations were more popular in advanced thyroid cancers and strongly correlated with poor clinical outcomes [6–8, 13]. *EIF1AX* gene was recently identified as a new thyroid cancer-related gene. *EIF1AX* mutations were strikingly enriched in poorly differentiated thyroid cancer (PDTC) and anaplastic thyroid cancer (ATC) and predicted for shorter survival in PDTC [14]. Distinct mutations on *EIF1AX* may be correlated with different tumor phenotypes [15]. However, the role of *TERTp* or *EIF1AX* mutations in follicular thyroid tumors hadn’t been fully investigated.

With the development of high-throughput sequencing technologies, next- generation sequencing (NGS)-based molecular testing is playing a vital role in diagnosis, prognosis and treatment monitoring [16]. For example, the identification of *BRAF* V600E from fine-needle aspiration specimens is used to diagnose papillary thyroid cancer (PTC) or PTC-derived anaplastic thyroid cancer [17]. The purpose of the study was to derive mutation profiles in a representative cohort of patients with FTA/FT-UMP/WDT-UMP/FTC using capture-based targeted sequencing with an 18-gene panel and to correlate the mutation profiles with clinicopathological features and prognosis.

## Materials and methods

### Study cases

One hundred and fifty-three cases initially diagnosed as FTA, atypical follicular thyroid adenoma (AFTA) and FTC between June 2010 and May 2015 were derived from the database of the Department of Pathology, Peking Union Medical College Hospital. All patients received surgical treatment. All corresponding archived hematoxylin and eosin-stained tumor sections were collected and independently re-evaluated by two experienced pathologists (Wu H and Ren X). The third pathologist (Liang Z) was available if necessary. Finally, 152 cases including 48 FTA, 17 WDT-UMP, 32 FT-UMP and 55 FTC were histologically verified based on the 2017 World Health Organization (WHO) classification of endocrine tumors. FTC was further classified into minimally invasive group, encapsulated angioinvasive group and widely invasive group. Hürthle cell tumors were integrated into subtypes of those tumors, although they were suggested as distinctive ones [2]. The American Joint Committee on Cancer (AJCC) stages were defined according to the 8th edition. Moreover, patient demographics and survival outcomes were obtained. The institutional review board of Peking Union Medical College Hospital approved the study, and because of its retrospective nature, written informed consent was waived.

### DNA extraction

Ten tumor sections (5 μm thick each) from qualified formalin-fixed, paraffin-embedded (FFPE) tissue blocks were mounted on slides. The tumor sections were deparaffinized using xylene, dehydrated step-wise with ethanol, and one of the slides stained with HE. An experienced pathologist (Wu H) examined and marked the target area of tumor cells on the slides. DNA was extracted using QIAamp DNA FFPE Tissue Kit (QIAGEN, California, USA) according to the manufacturer’s instructions. Quality control of DNA samples were evaluated using the Invitrogen Qubit 3 fluorometer (Thermo Fisher Scientific, California, USA).

### Library preparation

DNA fragmentation was performed using Covaris M220, followed by end repair, phosphorylation and adaptor ligation. Fragments of size 200–400 bp were selected by AMPure beads (Agencourt AMPure XP Kit, Beckman Coulter, California, USA) followed by hybridization with capture probes baits, hybrid selection with magnetic beads and PCR amplification. Subsequently, high-sensitivity DNA assay was performed to assess the quality and the size of all fragments. Indexed samples were sequenced on Nextseq500 sequencer (Illumina, Inc., California, USA) with paired-end reads using 18 thyroid cancer-related genes. The panel comprises 18 genes, which are closely relevant to the pathogenesis and development of thyroid cancer. The panel covers selected exons, introns or promoter regions of *TERT, EIF1AX, H/N/K-RAS, BRAF, TP53, PIK3CA, PTEN, GNAS, TSHR, CTNNB1, AKT1* and *ETV6*, and was designed to detect single nucleotide substitutions and small indels in all these genes. Additionally, the panel was capable of identifying large gene rearrangements at *RET, PPARG, ALK,* and *NTRK1.* Both known hotspot mutations and novel variants could be detected.

### NGS analysis

Sequencing data was mapped to the human genome (hg19) using Burrows-Wheeler Aligner 0.7.10. Local alignment optimization, variant calling and annotation were performed using GATK 3.2, MuTect, and VarScan. DNA translocation analysis was performed using both Tophat2 and Factera 1.4.3. Variants were filtered using the VarScan filter pipeline. According to the ExAC, 1000 Genomes, dbSNP, ESP6500SI-V2 database, variants with population frequency over 0.1% were grouped as common SNPs and removed. Remaining variants were annotated with ANNOVAR and SnpEff v3.6. To avoid errors related to sequencing or aligning, variants were filtered to retain only those covered by at least 100 reads, and Integrative Genomics Viewer (Broad Institute, USA) was employed to visualize variants aligned against the reference genome to confirm the accuracy of the variant calls by checking for possible strand biased and sequencing errors.

All variants were either confirmed somatic ones (as described in the Catalogue Of Somatic Mutations In Cancer (COSMIC) database [18]) or were predicted by three algorithms including SIFT, PolyPhen-2 and PROVEAN [19]. Mutations likely to result in altered protein function were described as deleterious, damaging, or probably damaging. Mutations shown simultaneously as tolerated, benign and neutral in the three algorithms, or mutations predicted as neutral in COSMIC database were excluded from further analysis. *H/N/K-RAS*, *PTEN*, *RET, BRAF*, *EIF1AX* and *TSHR* are known as early driver genes in thyroid carcinogenesis, and when the allelic frequency was ≥10%, the mutations were regarded as positive test results. Mutations in genes, such as *TP53*, *TERT, CTNNB1* and *PIK3CA* are developed subsequently in the process of carcinogenesis, and mutations were considered as positive ones if their allelic frequency was ≥5% [20].

### Statistical analyses

Data was presented either as frequencies and percentages, or as means and standard deviations or medians and interquartile range. Categorical variables were compared using either Pearson chi-square test or Fisher’s exact test. Continuous variables were compared using either the independent *t* test or one-way analysis of variance. Disease-free survival (DFS) was determined between the date of evaluation, and the date of recurrence or date of last known status. Survival curves were plotted with Kaplan-Meier method with log-rank statistics. Cox proportional hazards regression was used to assess the risk of recurrence. Statistical significance was defined as two-sided values of *P* <0.05. Statistical analyses were conducted with SPSS version 23.0 (SPSS Inc). Mutation plots were generated using Mutation Mapper tools, which are available at the cBioPortal [21, 22]. Graphic representations of gene mutations were performed on GraphPad Prism 7.00 (GraphPad Software).

## Results

### Mutation signature for distinguishing FTA, TT-UMP and FTC

**Fig. 1:**
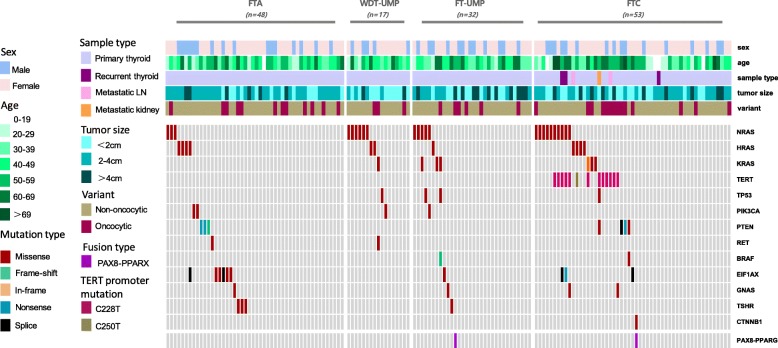
Clinicopathological features and mutation spectrum in 150 follicular thyroid tumors. Clinicopathological characteristics and genes were listed on the right of the mutation spectrum and color keys for them were shown on the left

We profiled 150 patients with follicular thyroid tumors, including 48 with FTA, 32 with FT-UMP, 17 with WDT-UMP and 53 with FTC (Two cases of FTC were deleted due to DNA sequencing failure) (Table 1). A total of 49% of patients (73/150) carried at least one mutation, involving 95 nonsilent somatic mutations spanning 14 genes (Fig. 1). Genetic alterations were summarized and compared. First, the rates of concurrent mutations were different among the four subtypes. Approximately, 2% FTA (1/48), 6% WDT-UMP (1/17), 13% FT-UMP (4/32) and 21% FTC cases (11/53) carried concurrent gene mutations. Three concurrent somatic mutations were only detected in patients with FTC (8%, 4/53). There was a significant difference about the rates of concurrent mutations in at least two genes among the four subtypes (*p* = 0.018). *H/N/K-RAS* were the most frequently co-mutated genes. Second, the predominant gene mutation feature was different. *TERTp* mutations were exclusively detected in FTC. A total of 80% *EIF1AX* exon 2 mutations (4/5) and 75% *TSHR* mutations (3/4) occurred in FTA, whereas 20% *EIF1AX* exon 2 mutations (1/5) and 25% *TSHR* mutations (1/4) occurred in FT-UMP. *KRAS* mutations and *TP53* mutations were only present in the borderline or malignant tumors. *H/N-RAS* mutations were detected in all four subtypes, but were most commonly found in WDT-UMP(*p* = 0.031). *BRAF* V600E and *RET* fusions were absent in the entire cohort. Overall, *TERTp*, *EIF1AX*, *TSHR*, *H/N/K-RAS* and *TP53* genes may be helpful to differentiate the subtypes of follicular thyroid tumors.

**Table 1:** Clinicopathological features of 150 patients with follicular thyroid tumors

	FTC, n (%)	FTA, n (%)	FT-UMP, n (%)	WDT-UMP, n(%)
Cases	53	48	32	17
Sex				
Male	16 (30)	16 (33)	12 (37)	5 (29)
Female	37 (70)	32 (67)	20 (63)	12 (71)
Age at diagnosis				
Mean year	46	45	46	44
Median (Quartiles)	50 (31–61)	47 (34–54)	44 (37–58)	43 (33–55)
Tumor size, cm				
Mean size	3.1	2.5	3.4	1.9
Median (Quartiles)	2.5 (1.5–4.0)	2.5 (1.8–3.0)	2.5 (1.4–5.4)	1.2 (1.0–2.7)
Variant type				
Hürthle cell tumor	14 (26)	12 (25)	6 (19)	2 (18)
Non-Hürthle cell tumor	39 (74)	36 (75)	26 (81)	14 (82)
Histologic type ^a^				
Minimally invasive	25 (47)			
Encapsulated angioinvasive	15 (28)			
Widely invasive	13 (25)			
Lymph node metastasis				
Present	5 (9)	–	–	–
Distant metastasis				
Present	2 (4)	–	–	–
AJCC stage				
I~II	51 (96)	–	–	–
III~IV	2 (4)	–	–	–
Follow-up time				
Median (Quartiles)	56 (46–65)	55 (42–65)	55 (47–60)	55 (29–74)
Disease Persistence/Recurrence				
Yes	8 (15)	0 (0)	0 (0)	0 (0)
No	45 (85)	48 (100)	32 (100)	16 (100)

^a^, based on the 2017 World Health Organization classification of endocrine tumors; Abbreviations: *FTC* follicular thyroid carcinoma, *FTA* follicular thyroid adenoma, *FT-UMP* follicular tumor of uncertain malignant potential, *WDT-UMP* well-differentiated tumor of uncertain malignant potential, *AJCC* American Joint Committee on Cancer, 8th edition staging;

### H/N/K-RAS, TERTp and EIF1AX mutations in follicular thyroid tumors

**Fig. 2:**
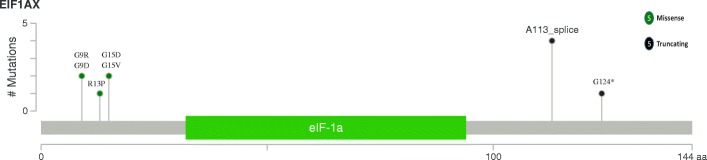
*EIF1AX* mutations detected in our cohort. The colored box depicts the functional domain along the protein. The location of the circle specifies the mutation site. A patient is represented by a circle. Green circles represent missense mutations and black circles represent truncating mutations. The number of circles in a lollipop represents the number of patients harboring the specific variant

We identified frequent *H/N/K-RAS* mutations in all subtypes (28%, 42/150). *HRAS* mutations were detected in 8% FTA, 12% WDT-UMP, 3% FT-UMP and 8% FTC cases. They were located at codon 61/13. *NRAS* mutations were identified in 6% FTA, 35% WDT-UMP, 16% FT-UMP and 19% FTC cases. They were all located at codon 61. *KRAS* mutations were found in 6% WDT-UMP, 9% FT-UMP and 6% FTC. They were mainly located at codon 61 (57%, 4/7). *H/N/K-RAS* genes were mutated exclusively except for one case of FT-UMP exhibiting concurrent *KRAS* and *NRAS* mutations. Our results showed *TERTp* mutations were exclusively detected in 25% of patients with FTC (13/53). C228T was the predominant form of mutation, accounting for 92% of detected mutations (12/13) and the other was C250T. *EIF1AX* mutations were observed in FTC, FT-UMP and FTA. *EIF1AX* mutations were identified in 6% FTC cases (3/53), and all located at intron 5/exon 6 (2 *EIF1AX* A113_splice and 1 G124* mutation). Two of three *EIF1AX* mutations were concurrent with *NRAS* mutations. *EIF1AX* mutation were identified in 13% FTA (6/48), 4 *EIF1AX* exon 2 and 2 A113_splice mutations. One *EIF1AX* A113_splice mutation coexisted with *HRAS* mutation. More details were shown in Fig. 2. In order to have an overall understanding of *EIF1AX* mutation detected in thyroid tumors, we also analyzed the occurrence of EIF1AX mutations in the COSMIC database [23], cBioPortal for cancer genomics database [24] and previous studies [14, 15, 25–29] (Table 2). *EIF1AX* intron5/exon6 mutations occurred in benign or malignant thyroid tumors. Concurrent *EIF1AX* exon 2 and *H/N/K-RAS* mutations were only observed in PDTC and ATC. *EIF1AX* exon 2 mutations without *H/N/K-RAS* mutations were only detected in the benign lesions, borderline lesions and PTC.

**Table 2:** *EIF1AX* mutations were summarized based on our results, COSMIC database, cBioPortal for cancer genomics database and previous studies (references14–15, 23–27)

Disease	Cases	*EIF1AX* exon2 mutations		*EIF1AX* intron5/exon6 mutations ^a^	
		Total cases	With *RAS* Mut	Total cases	With *RAS* Mut
FA/HN	15	10	0	5	2
FT-UMP	1	1	0	0	0
PTC	10	4	0	6	4
FTC	7	0	0	7	4
PDTC	13	3	3	10	8
ATC	14	5	5	9	9

^**a**^, A113_splice mutation and G124* mutations

Abbreviations: *FA/HN* follicular adenoma/hyperplastic nodules, *FT-UMP* follicular tumor with uncertain malignant potential, *PTC* papillary thyroid carcinoma, *FTC* follicular thyroid carcinoma, *PDTC* poorly differentiated thyroid carcinoma, *ATC* anaplastic thyroid carcinoma

In FTC, we illustrated that patients with co-occurring *TERTp* and *H/N/K-RAS* mutations were older and presented with advanced disease (*p* = 0.013, *p* = 0.015, respectively), compared to other patients (Table 3). Similarly, we clarified *EIF1AX* mutations were correlated with advanced disease (*p* = 0.032), with 2% of mutation rate in stage I FTC (1/44), 14% of mutation rate in stage II FTC (1/7), 100% of mutation rate in stage III FTC (1/1).

**Table 3:** Association of *TERTp* and *H/N/K-RAS* mutations with clinicopathological featurs in 53 FTC patients

Variations	*H/N/K-RAS- TERTp-*	*H/N/K-RAS+ TERTp-*	*H/N/K-RAS- TERTp+*	*H/N/K-RAS+ TERTp+*	*P*
Cases	30	10	6	7	
Sex					0.406
Male	8 (27)	2 (20)	2 (33)	4 (57)	
Female	22 (73)	8 (80)	4 (67)	3 (43)	
Age, years					0.013
Median ± SD	43 ± 3	33 ± 5	59 ± 2	66 ± 3	
Tumor size (cm)					0.764
<2.0	8 (27)	3 (30)	3 (50)	3 (43)	
2.0–4.0	16 (53)	5 (50)	1 (17)	3 (43)	
>4.0	6 (20)	2 (20)	2 (33)	1 (14)	
Lymph node metastasis					0.271
Yes	2 (7)	1 (10)	0	2 (29)	
No	28 (93)	9 (90)	6 (100)	5 (71)	
Distant metastasis					0.057
Yes	0	0	1 (17)	1 (14)	
No	30 (100)	10 (100)	5 (83)	6 (86)	
AJCC Stage					0.015
I	27 (90)	10 (100)	4 (67)	3 (43)	
II	3 (10)	0	2 (33)	2 (29)	
III	0	0	0	1 (14)	
IV	0	0	0	1 (14)	
Histologic type					0.681
Minimally invasive	16 (53)	5 (50)	2 (33)	2 (28)	
Encapsulated angioinvasive	7 (23)	4 (40)	2 (33)	2 (28)	
Widely invasive	7 (23)	1 (10)	2 (33)	3 (43)	
Disease recurrence/persistence ^a^					0.026
Yes	2 (7)	1 (10)	2 (29)	3 (50)	
No	28 (93)	9 (90)	4 (71)	3 (50)	

^a^, fifty-two patients were available to evaluate the disease recurrence/persistence status, the other patient was lost to follow-up

Abbreviations: *TERTp*, *TERT* promoter, *FTC* follicular thyroid carcinoma, *AJCC* American Joint Committee on Cancer, 8th edition staging, *P P*-value, *median ± SD* median ± standard deviation

### Genetic comparison of Hürthle cell tumors and non-Hürthle cell tumors

Hürthle cell tumors were separated from the follicular tumors for their distinct genetic profiles in the 2017 WHO classification of endocrine tumors. In our cohort, 35 Hürthle cell tumors and 115 non-Hürthle cell tumors cases were identified. Genetic comparison of the two types of thyroid tumors were performed. First, *TERTp* and *KRAS* mutations were identified in 17 and 11% Hürthle cell tumors, respectively, whereas it was 6 and 3% in non-Hürthle cell tumors, respectively. Second, *NRAS* mutations tended to be more popular in non-Hürthle cell tumors with incidence of 19%, compared with that of 6% in Hürthle cell tumors. Six co-occurring *TERTp* and *H/N-RAS* mutations all occurred in non-Hürthle cell tumor, whereas no such alteration was observed in Hürthle cell tumor.

### Survival analysis

In our cohort, survival outcomes could be evaluated in 94% FTA (45/48), 100%WDT-UMP (17/17), 91% FT-UMP (29/32) and 98%FTC (52/53). Only one FTC patient with initial bone metastasis maintained disease persistence and experienced disease-specific death. Another FTC patient with kidney metastasis at diagnosis was lost to follow-up. Collectively, seven patients with FTC exhibited disease recurrence in the first four years, comprising four local recurrence, one reginal recurrence and two distant recurrences (both spread to lungs). In contrast, no disease recurrence was observed in FTA, FT-UMP or WDT-UMP patients. We performed a detailed investigation regarding the correlation of clinicopathological and genetic variables and disease-free survival (Table 4). Based on the univariate Cox analysis, predictors of shorter DFS were lymph node metastasis (95% confidence interval (CI) 1.929–39.145, *p* = 0.005) advanced disease (95% CI 1.646–152.169; *p* = 0.017), widely invasive histologic subtype (95% CI 1.084–21.761, *p* = 0.039), *EIF1AX* mutations (95% CI 1.887–52.892; *p* = 0.007), and *TERTp* mutations (95% CI 1.254–25.298; *p* = 0.024). In multivariable analysis, no independent factors were identified.

**Table 4:** Association of clinicopathological and genetic characteristics with disease- free survival in 51 FTC

		Univariate		Multivariate	
Variations	Cases	HR(95% CI)	*P*	HR(95% CI)	*P*
Sex			0.138		
Male	16	1.000(reference)			
Female	35	0.321 (0.072–1.439)			
Age at diagnosis, years			0.425		
<55	29	1.000(reference)			
≥ 55	22	1.840 (0.411–8.234)			
Tumor size, cm			0.762		
< 2.0	16	1.000(reference)			
2.0~4.0	24	0.637 (0.128–3.158)			
> 4.0	11	0.471 (0.049–4.531)			
Lymph node metastasis			0.005		0.442
No	46	1.000(reference)		1.000(reference)	
Yes	5	8.689 (1.929–39.145)		2.632 (0.224–30.951)	
AJCC Stage			0.017		0.770
I + II	50	1.000(reference)		1.000(reference)	
III + IV	1	15.827 (1.646–152.169)		1.742 (0.043–71.363)	
Histologic type^a^			0.039		0.182
Minimally invasive /Encapsulated angioinvasive	39	1.000(reference)		1.000(reference)	
Widely invasive	12	4.856 (1.084–21.761)		4.125 (0.515–33.049)	
Hürthle cell tumors			0.992		
No	37	1.000(reference)			
Yes	14	0.991 (0.192–5.111)			
EIF1AX mutation			0.007		0.366
WT	48	1.000(reference)		1.000(reference)	
Mut	3	9.989 (1.887–52.892)		3.998 (0.198–80.778)	
H/N/K-RAS mutation			0.393		
WT	44	1.000(reference)			
Mut	7	1.920 (0.430–8.586)			
*TERTp* mutation			0.024		0.162
WT	40	1.000(reference)		1.000(reference)	
Mut	11	5.633 (1.254–25.298)		3.841 (0.583–25.307)	

^a^based on the 2017 World Health Organization classification of endocrine tumors; Abbreviations: *WT* wild-type, *FTC* follicular thyroid carcinoma, *AJCC* American Joint Committee on Cancer, 8th edition staging, *HR* hazard ratio, *95%CI* 95% confidence interval, *P P*-value

**Fig. 3:**
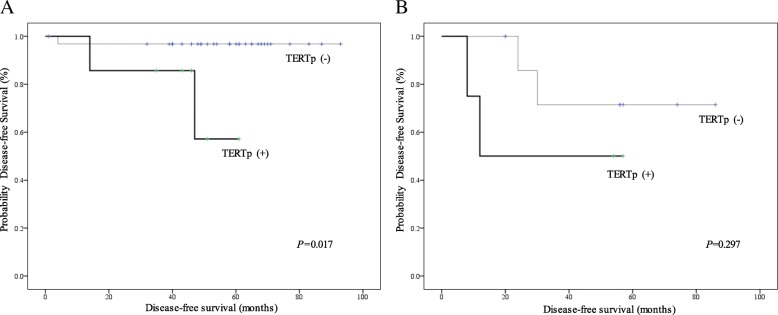
Kaplan-Meier curves of disease-free survival by *TERTp* mutational status in patients with FTC. Results from the analysis of patients with (**a**) minimally invasive /encapsulated angioinvasive histologic type and (**b**) widely invasive histologic type

We further analyzed the impact of *TERTp* mutation status on survival outcome in FTC patients with three histologic types. In patients with minimally invasive/encapsulated angioinvasive FTC, *TERTp* mutations contributed to shorter DFS than *TERTp* wild-type did (*p* = 0.017), whereas *TERTp* mutations were not correlated with shorter DFS in patients with widely invasive FTC(*p* = 0.297). Kaplan-Meier survival analysis and compared statistically using the log-rank test were performed (Fig. 3).

## Discussion

FTA, FT-UMP, WDT-UMP and FTC can hardly be precisely distinguished based on histopathological features, and the differential diagnosis may sometimes be arbitrary and difficult. Molecular diagnostic tools would may help to determine accurate diagnosis by exploring the genetic characteristics. Our study used capture-based targeted sequencing with an 18-gene thyroid tumor panel to detect and quantify genetic alterations associated with these tumors, aiming to derive potential diagnostic and prognostic biomarkers. Our results showed that *TERTp*, *EIF1AX*, *TSHR*, *H/N/K-RAS* and *TP53* genes may be potential diagnostic or prognostic markers in follicular thyroid tumors. In FTC, *TERTp* mutations were correlated with shorter DFS in patients with minimally invasive/encapsulated angioinvasive histologic feature, but not in patients with widely invasive histologic feature.

Given that the molecular similarities concerning *TERTp* mutations or aberrations between FTC and FT-UMP groups, and that the *TERTp-*mutated FTA developed a scar recurrence and died of FTC, *TERTp* mutational testing is thought to detect malignant potential in follicular thyroid tumors [6, 11]. Our results showed patients with FT-UMP or FTA carried no *TERTp* mutations, in agreement with previous studies, which reported that normal tissues and benign lesions of the thyroid carried no *TERTp* mutations [7–10, 12, 13]. The reasons may be due to the rarity of *TERTp* mutations in non-malignant follicular thyroid tumors, inadequate tumor sampling or interobserver variability between pathologists in diagnosing thyroid borderline tumors [3, 26, 30]. The interobserver variability may be affected by the differences in clinical practice in the different regions. Borderline tumors were often treated as thyroid carcinoma in western clinical practices, whereas according to Asian practice they were handled as if benign tumors [30]. Additional international cooperation might be needed to further clarify the role of *TERTp* mutations in FTC tumorigenesis.

Interestingly, our further analysis showed that *TERTp* mutations were significantly correlated with shorter DFS in patients with minimally invasive/encapsulated angioinvasive FTC, but not in patients with widely invasive FTC. Recently, Bournaud et al. also reported that *TERTp* mutations might be helpful to better define the prognosis of localized thyroid cancer without aggressive histology. However, only a few cases of FTC were included in their study [31]. It provides a new perspective on the prognostic value of *TERTp* mutations in FTC. We also clarified that FTC patients with co-occurring *TERTp* and *H/N/K-RAS* mutations were older, presented with advanced disease, and had higher disease recurrent/persistent rate, compared with other FTC patients. Moreover, the only deceased patient in our cohort was the patient with FTC carrying concurrent *TERTp* and *NRAS* mutations. Therefore, the synergistic role of *TERTp* and *H/N/K-RAS* mutations might exist in FTC, which is in agreement with literature findings [13, 32, 33].

Our results showed that *NRAS* mutations were more detected in non-Hürthle cell tumors, whereas *KRAS* mutations were more common in Hürthle cell tumors. That is in accordance with the study by Radkay et al. [34], where compared to *H/N-RAS* mutation, *KRAS* mutation was more common in thyroid nodules with oncocytic change. Coexisting *TERTp* and *H/N-RAS* mutations were all detected in non-Hürthle cell cancer, whereas *TERTp* without *H/N-RAS* mutations occurred in Hürthle cell cancer. These results indicated biologically distinct genetic features between non-Hürthle and Hürthle cell tumors.

Co-occurrence of *EIF1AX* and *H/N/K-RAS* mutations is correlated with tumor aggressiveness, especially when it is the A113_splice mutations for *EIF1AX* gene [15, 25]. That is consistent with our results that *EIF1AX* mutations in FTC were located at intron 5/exon 6, correlated with advanced disease, and coexisted with *NRAS* mutation. Moreover, we also identified one *EIF1AX* G124* nonsense mutation in FTC, which was only previously reported in a case of PTC (The Cancer Genome Atlas-EM-A3ST-01). *EIF1AX* exon2 mutations without *H/N/K-RAS* mutations, which existed in benign lesions, borderline lesions and PTC, but not in FTC, PDTC and ATC, might be endowed as a diagnostic marker.

*BRAF* V600E mutation was absent in WDT-UMP, which is consistent with previous studies [2, 35]. Recently, Amendoeira et al’s review summarized the genotypic abnormalities of NIFTP in several studies. From their reports, *H/N/K-RAS* mutations were detected in 44.9% NIFTP cases (119/265) and more than half of the previous studies detected no *BRAF* V600E mutation in NIFTP cases [36]. The research of Kim et al. also showed a similar *H/N/K-RAS* mutation rate (47%), and absence of *BRAF* V600E mutation in their NIFTP series [37]. In the present study, we identified *H/N/K-RAS* mutations and *BRAF* mutations respectively in 53 and 0% of the WDT-UMP cases. The result suggests that WDT-UMP might be genetically similar to non-invasive follicular thyroid neoplasm with papillary-like nuclear features (NIFTP). A previous report identified a relatively low rate of *H/N/K-RAS* mutations in WDT-UMP [36]. One plausible reason for the difference is that the majority of our WDT-UMP cases had PTC-type nuclear alteration, which histologically overlapped with NIFTP. However, given the limitation of our single institution experience, the results need further validation in multicenter studies with large cohorts of patients.

*TP53* mutations were not identified in FTA group but in borderline/malignant tumors. In the *TP53*-mutated WDT-UMP, the tumor size was 4 cm, much larger than the median size of 1.2 cm in WDT-UMP group. Those suggested a possible subgroup of *TP53*-mutated tumors, and its potential role in progression to a more aggressive phenotype that has not yet fully manifested in thyroid tumors [37, 38]. Although *TP53* mutations were more found in FT-UMP than in FTC, there was no significant difference regarding the prevalence of *TP53* mutations. That may result from the small sample size in our cohort. Further studies clarifying the role of *TP53* mutations in patient diagnosis and prognostic significance in larger sample size should be organized [37].

The frequency of *PAX8-PPARG* fusion in our cohort was much lower than 30–60% noted in reports from other countries [39]. Kunio et al. also detected low frequency of *PAX8-PPARG* fusion (1/24, 4%) in FTC, suggesting a possible distinct genetic feature in FTC in Japanese patients due to the high iodine intake from a typical Japanese diet [40]. Chinese population has an iodized salt diet, for the iodized salt policy has been implemented by Chinese government since 1995. It implicates that the demographic background may have an influence on the rate of *PAX8-PPARG* fusion.

In summary, *TERTp*, *EIF1AX, TSHR, H/N/K-RAS* and *TP53* mutations may have diagnostic and prognostic potential in follicular thyroid tumors. *TERTp* mutations may be indicative of poor outcome in FTC patients with minimally invasive/ encapsulated angioinvasive histological features.

## Abbreviations

AFTA: Atypical follicular thyroid adenoma
AJCC: The American Joint Committee on Cancer
ATC: Anaplastic thyroid cancer
COSMIC: The Catalogue Of Somatic Mutations In Cancer
DFS: Disease-free survival
FFPE: Formalin-fixed, paraffin-embedded
FTA: Follicular thyroid adenoma
FTC: Follicular thyroid cancer
FT-UMP: Follicular tumors of uncertain malignant potential
NGS: Next- generation sequencing
PDTC: Poorly differentiated thyroid cancer
PTC: Papillary thyroid cancer
*TERTp*: *TERT* promoter
TT-UMP: Thyroid tumors with uncertain malignant potential
WDT-UMP: Well-differentiated tumors of uncertain malignant potential
WHO: World Health Organization
